# Agenesis of Permanent Mandibular Central Incisors: A Concordant Condition in Siblings

**DOI:** 10.5005/jp-journals-10005-1337

**Published:** 2016-04-22

**Authors:** Pawwan Kumar Kagitha, Srinivas Namineni, Abhinaya Reddy Tupalli, Santhosh Kumar Challa

**Affiliations:** 1 Postgraduate Student, Department of Pediatric Dentistry, Sri Sai College of Dental Surgery, Vikarabad, Telangana, India; 2 Professor and Head, Department of Pediatric Dentistry, Sri Sai College of Dental Surgery, Vikarabad, Telangana, India; 3 Reader, Department of Pediatric Dentistry, Sri Sai College of Dental Surgery, Vikarabad, Telangana, India; 4 Reader, Department of Pediatric Dentistry, Sri Sai College of Dental Surgery, Vikarabad, Telangana, India

**Keywords:** Agenesis, Central incisor, Concordance, Hypodontia, Siblings.

## Abstract

Concordance is an identical expression of phenotype in two related individuals. Concordance expression of hypodontia is an uncommon condition where associated individuals are affected with exactly similar kind and number of missing teeth. There is very limited documentation of this condition either in twins or in siblings, and literature shows paucity of data with regard to this anomaly. To the best of our knowledge, there is only one such case reported in the literature, which has actually showed similar missing lower central incisors in siblings. This report presents a case of two girl siblings aged 11 and 13 years with congenital bilateral missing of permanent mandibular central incisors, which is an absolute concordant condition. Apart from discussing etiology, clinical implications and management, this article highlights the significance of concordant and discordant condition of hypodontia and expression of this condition in twins and siblings.

**How to cite this article:** Kagitha PK, Namineni S, Tupalli AR, Challa SK. Agenesis of Permanent Mandibular Central Incisors: A Concordant Condition in Siblings. Int J Clin Pediatr Dent 2016;9(1):74-77.

## INTRODUCTION

The congenital absence of one or more teeth, either primary or permanent, is referred to as hypodontia. Varied findings have been reported concerning the percentage of the population affected by this condition. In addition, the literature supporting the tooth or teeth that are most frequently missing have also been inconsistent. Among various populations, prevalence of hypodontia varies as 4.19% in India to 11.3% in Ireland. The most frequently missing permanent teeth, excluding third molars, are the mandibular second premolar (11.3%) followed by mandibular incisor (6.9%) and maxillary lateral incisor (6.5%).^1^

Most of the studies have shown the congenital absence affecting a single tooth, but not both, in a pair, suggesting its occurrence being unilateral.^2^ However, there are few reports of hypodontia where teeth were missing bilaterally. As far as bilateral agenesis is concerned, the mandibular central incisors are frequently prone and it was first reported by Newman in 1967.^3^ Reports suggesting the bilateral agenesis of teeth in siblings are unusual and rare. Fukawa^4^ first documented this condition in siblings in 1993.

## CONCORDANCE AND DISCORDANCE

Concordance is an identical expression of phenotype in two related individuals, usually seen in monozygotic twins due to similar genetic makeup. Discordance is a condition, unlike concordance, where expression of phenotype is uneven in two associated individuals. This condition is frequently seen in dizygotic twins and siblings as they both have dissimilar genetic characteristics.^5^ Zeiger and Winkler in 1931^6^ cited two cases of identical twins in which the hypodontia was concordant and in another case of fraternal twins in which it was discordant. Boruchov and Green in 1979^5^ cited large-scale reports on hypodontia concerning the concordance and discordance distributions for missing teeth in monozygotic, dizygotic twin pairs and siblings. They concluded that discordant hypodontia (for any or same tooth) is a more favorable phenomenon even in monozygotic twins when compared with concordant hypodontia. This negative tendency toward concordant expression might be due to epigenetic influences and environmental factors overshadowing the hereditary elements, resulting in altered expression of hypodontia in associated individuals. To date, however, there is limited documentation on concordance condition of hypodontia either in twins or siblings and literature shows paucity of data in this regard.

This article reports a case of absolute concordant condition of hypodontia in siblings affecting bilateral permanent mandibular central incisors. The article focuses on etiology, sequel and various available treatment modalities with respect to mandibular incisor agenesis.

## CASE REPORT

**Figs 1A and B:**
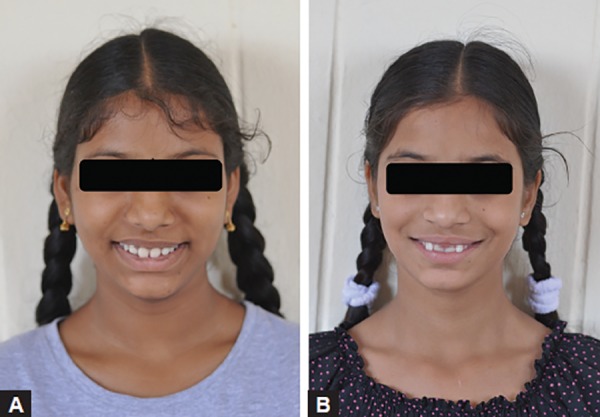
(A) Pretreatment facial photographs of sibling A and (B) pretreatment facial photographs of sibling B

**Figs 2A and B:**
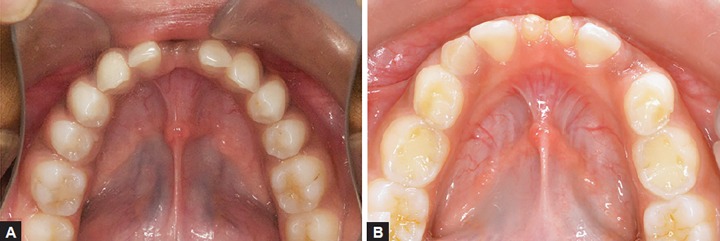
(A) Intraoral photograph of sibling A, showing retained left deciduous mandibular central incisor and missing permanent mandibular central incisors and (B) intraoral photograph of sibling B, showing retained deciduous mandibular central incisors

**Figs 3A and B:**
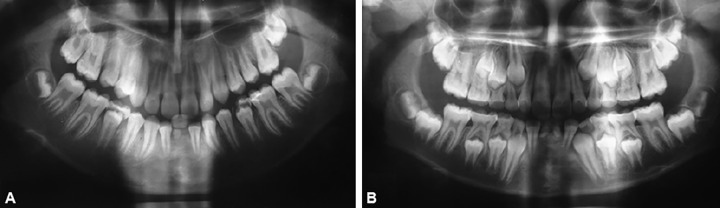
(A) Panoramic radiograph of sibling A, revealing retained left deciduous mandibular central incisor and missing permanent mandibular central incisors and (B) panoramic radiograph of sibling B, revealing retained deciduous mandibular central incisors

Two girl siblings aged 11 and 13 years (Figs 1A and B) reported to the Sri Sai College of Dental Surgery, Vikarabad, complaining of retained lower anterior primary teeth. Upon review of the family history, it was known that the children were born full term to parents of a nonconsanguineous marriage. Medical history and general examination of both the children gave no significant data. Intraoral examination of the elder sibling (Fig. 2A) showed the presence of retained left primary mandibular central incisor and clinically missing right permanent mandibular central incisor. The retained deciduous incisor was attrited and mobile. The other sibling (Fig. 2B) presented with retained left and right primary mandibular central incisors, which were clinically sound with no mobility. There was no history of previous infection, metabolic disorders and trauma. All other teeth were normal in shape, size and color. Both of them did not express features of any syndromes. A panoramic radiograph was taken which revealed the congenital absence of both the permanent mandibular central incisors in both the siblings (Figs 3A and B).

**Fig. 4:**
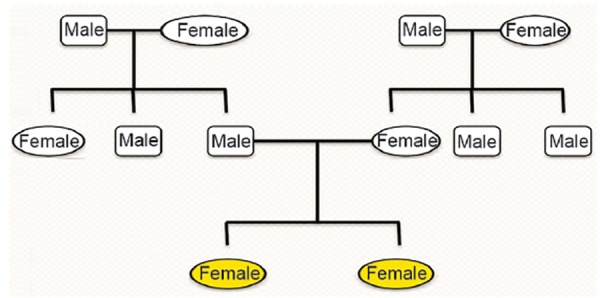
Pedigree chart for three generations of respective family showing no significant history

Pedigree charting was then done on their family for three generations and there were no signs of hypodontia or any other anomalies or syndromes (Fig. 4).

**Figs 5A and B:**
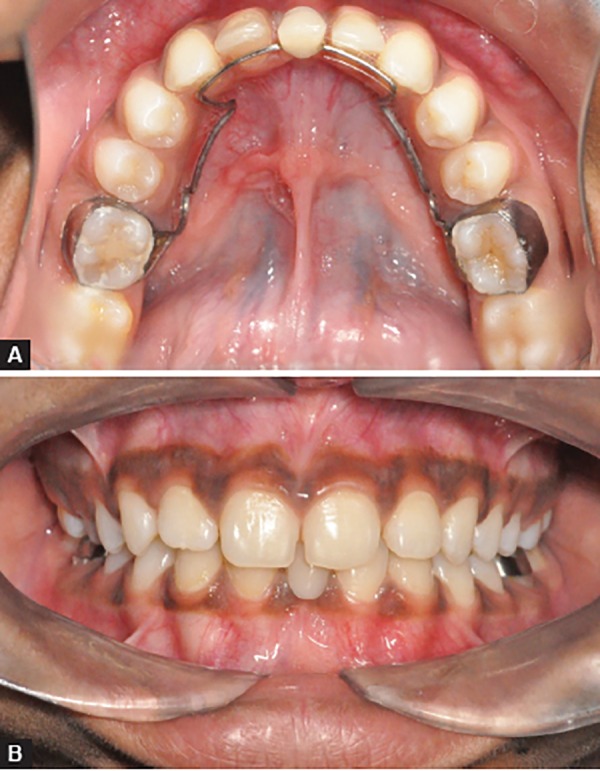
(A) Posttreatment occlusal view of sibling A, showing lingual arch-supported acrylic prosthesis and (B) posttreatment frontal view of sibling A, showing acrylic prosthesis

Parent and patient were informed about the condition and various possible treatment options were elucidated. Extraction of retained primary central incisor was done in the elder sibling and fixed functional space maintainer was planned, i.e., lingual arch with anterior acrylic prosthesis (Figs 5A and B). In the younger sibling, no treatment was done as the retained deciduous central incisors exhibited no mobility and showed minimal resorption. Both the children were put under follow-up and planned for fixed permanent prosthesis once cessation of growth occurs.

## DISCUSSION

The dissimilar expression of hypodontia in siblings is possible but identical expression, i.e., concordant expression of hypodontia, is quite surprising inspite of the diverse genetic makeup along with varied environmental influences in two different individuals. Most investigations have reported that concordant phenotypic expression is a result of an expression of the same genotype rather than any environmental influences. This is commonly seen in monozygotic twins where if one of the twin in a pair has a trait, the risk that the other twin expressing the same phenotype is increased by 89%. On the contrary, in dizygotic twins and siblings, the risk of the other being affected is reduced to zero.^7^

In the present case report, both the siblings were affected with identical phenotypic expression of hypo-dontia, in spite of the differences in their genetic and environmental setup. This type of concordant expression in dissimilar siblings is unusual and this has not been documented before.

A specified set of homeobox genes including MAX1, PAX9, and AXIN2 program the development of human dentition where any mutations or alterations in signaling of these genes lead to agenesis of the respective tooth, it being a mandibular incisor in this case. Pani^8^ has reported that homeobox gene AXIN2 is implied to be involved with incisor agenesis but its mode of transmission is still left uncertain.

Environmental factors too pool with genetical aspects leaving a wide undefined area where etiology for concordant condition of hypodontia and pattern of incisor agenesis is left unclear. For instance, if a trait is determined genetically regardless of the birth rank, each of the siblings should have an equal opportunity of inheriting it. If it can be shown that the firstborn children have the trait expressed more often, birth trauma can be suspected as a possible cause. Likewise, if it is shown that the last-born children have the trait more often, the increasing age of the mother might be showing its effect.^5^

Mandibular incisor agenesis is distinct from other hypodontia conditions as it has an influence on esthetics affecting the child’s physical and emotional status and has a significant impact on the growth of the mandibular symphysis and maturation of its skeletal pattern. Although closure of the mandibular symphysis occurs in the 1st year of life, growth modifications in the anterior region are seen up to eruption of the permanent canines. Growth at symphyseal region and mandibular bone height is well associated with bone apposition at the dentoalveolar complex, especially during tooth eruption. Hence, patients with absence of mandibular central incisors exhibit significantly smaller mandibular symphysis area and greater retroclination of the mandibular alveolar bone than the normal patients. Agenesis of mandibular incisors also results in altered muscular forces due to imbalance between tongue and lip pressure that further deteriorates occlusal discrepancies like class II div I, anterior deep bite and reduced lower facial height.^9^ Insignificant volume of alveolar bone and smaller symphyseal region have influence on placement of future endosseous implants.^10^

Treatment of mandibular incisor agenesis requires an interdisciplinary approach that includes a team of a pediatric dentist, a prosthodontist, an orthodontist and an oral and maxillofacial surgeon. The treatment objectives include preservation of space, maintenance of alveolar integrity and prosthetic replacement of missing teeth to improve the function and enhance the esthetics. This can be achieved either with a removable or a fixed functional space maintainer. In the present case, fixed functional space maintainer was preferred over a removable appliance as the frequency of changing the appliance can be reduced keeping in view the growing age of the patient. A lingual arch with an acrylic prosthesis was given and the patient will be followed up until the growth ceases for future permanent prosthesis.

Implant-supported prosthesis in growing patients is the latest treatment modality which is more favorable in anterior mandible as the symphyseal closure is completed at 1 year of age and transverse growth is almost completed after eruption of permanent canines. In severe and syndromic hypodontia cases, benefits outweigh the disadvantages of implants in the growing patient, and literature supports placement of implants in these patients. In the present case, hypodontia being expressed only in missing mandibular central incisors, implant is not a recommended option as residual growth may lead to complications like infraocclusion of implant, vertical discrepancy of gingival margins, tipping of adjacent teeth, crossbite and loss of occlusal integrity.^10^

## CONCLUSION

The case reported here is a concordant expression of bilateral agenesis in siblings, a very rare condition. This article has reviewed etiological factors, inheritance and sequelae with respect to mandibular incisor agenesis that aids in understanding the basic concepts of concordant expression of hypodontia, its implications and treatment options that will enable pediatric dentists to address the patient’s esthetic and functional requirements over a long phase.
